# Transcranial magnetic stimulation and ketamine: implications for combined treatment in depression

**DOI:** 10.3389/fnins.2023.1267647

**Published:** 2023-10-26

**Authors:** Weronika Dębowska, Magdalena Więdłocha, Marta Dębowska, Zuzanna Kownacka, Piotr Marcinowicz, Agata Szulc

**Affiliations:** ^1^Department of Psychiatry, Faculty of Health Sciences, Medical University of Warsaw, Warsaw, Poland; ^2^KeyClinic, Warsaw, Poland; ^3^MindHealth, Warsaw, Poland

**Keywords:** depressive disorders, treatment-resistant depression, transcranial magnetic stimulation, TMS, rTMS, ketamine, combined TMS and ketamine, neuroplasticity

## Abstract

Drug-resistant mental disorders, particularly treatment-resistant depression, pose a significant medical and social problem. To address this challenge, modern psychiatry is constantly exploring the use of novel treatment methods, including biological treatments, such as transcranial magnetic stimulation (TMS), and novel rapid-acting antidepressants, such as ketamine. While both TMS and ketamine demonstrate high effectiveness in reducing the severity of depressive symptoms, some patients still do not achieve the desired improvement. Recent literature suggests that combining these two methods may yield even stronger and longer-lasting results. This review aims to consolidate knowledge in this area and elucidate the potential mechanisms of action underlying the increased efficacy of combined treatment, which would provide a foundation for the development and optimization of future treatment protocols.

## 1. Introduction

Depression is a chronic condition, whose symptoms include lowered mood and motivation. Left untreated, depression may lead to disability or death. Of the 280 million people suffering from depression worldwide, up to 35% are resistant to currently available drugs, which raises the necessity of developing novel treatment methods (Global Health Data Exchange, 2023) in the hope of alleviating symptoms in this group of patients. Among novel methods, biological interventions have shown promising results in the treatment of depression. Such methods include a variety of neurostimulation techniques, primarily: electroconvulsive therapy (ECT), deep brain stimulation (DBS), and transcranial magnetic stimulation (TMS). Additionally, psychotherapy is a highly recommended intervention, independent of the implemented treatment modality (McPherson and Senra, 2022). For a current review that comprehensively assesses the efficacy of various methods in the treatment of TRD, refer to McIntyre et al. (2023).

Transcranial magnetic stimulation (TMS) is the least invasive neurostimulation method and has the fewest side effects (O’Reardon et al., 2007). TMS induces a current in the cerebral cortex through the use of electromagnetic impulses. This is effective in approximately 30 to 90% of patients diagnosed with treatment-resistant depression (TRD), depending on the protocol used, and has almost no side effects (Rossi et al., 2009, 2021; Lefaucheur et al., 2020). TMS as a brain stimulation and potential therapeutic method was introduced in 1985, but in the field of psychiatry, it is still being perceived as innovative (Barker et al., 1985; Matsuda et al., 2021). The first TMS protocol was officially approved by the Food and Drug Administration (FDA) as a therapeutic modality for the treatment of depression in 2008 (Horvath et al., 2010).

Nevertheless, modern psychiatry still finds itself in need of novel therapeutic solutions. In recent years, ketamine—a substance known to medicine for several decades—has emerged as a promising alternative method for the treatment of depression. Ketamine is an antagonist of N-methyl-D-aspartate (NMDA) receptors primarily used in anesthesia. However, in subanesthetic doses, it exhibits rapid antidepressant, anxiolytic, and analgesic properties. In the treatment of depression, it is effective in up to 70% of patients, depending on the protocol used. Furthermore, it is the very first fast-acting antidepressant whose therapeutic effect may be observed already after the first dose (Smith-Apeldoorn et al., 2022). The FDA approved (S)-ketamine (an enantiomer) in the form of a nasal spray for TRD treatment in 2019. Although intravenous ketamine is more effective than (S)-ketamine, it is still being used as an off-label treatment (Berman et al., 2000; Marcantoni et al., 2020; Bahji et al., 2021). Despite being innovative and yielding rapid improvement, this method still leaves approximately 30% of patients without the desired outcome (Smith-Apeldoorn et al., 2022).

This review aims to offer valuable insights into the rationale behind utilizing a combination of TMS and ketamine as a more effective treatment strategy, which could improve symptoms in patients suffering from treatment-resistant depression.

## 2. TMS and ketamine

There are several potential mechanisms through which the combination of TMS and ketamine may interact, leading to a mutually reinforcing effect. To discuss potential synergy between TMS and ketamine, each method will be described individually, accompanied by a thorough analysis of the essential mechanisms of their actions.

### 2.1. Transcranial magnetic stimulation in the treatment of depressive disorder

Since the invention of TMS almost 40 years ago, multiple scientific articles have investigated its potential as a therapeutic tool and its impact on brain neuroplasticity. The basic principle of TMS is based on generating an electromagnetic pulse with a coil applied to the head. The pulse penetrates the skin, skull, meninges, and cerebrospinal fluid, and reaches the cerebral cortex. A single pulse causes a brief excitation of the stimulated region. For example, delivering such a pulse to the representation of the hand in the primary motor cortex (M1) can evoke a contraction of finger muscles in the contralateral hand; a similar pulse delivered to the primary visual cortex, may cause the subject to experience phosphenes (Amassian et al., 1998).

When pulses are applied in a repetitive manner (repetitive TMS, rTMS), their frequency can affect cortical activity in two ways. Low-frequency stimulation (lf-rTMS; 1 Hz or less) induces long-term depression (LTD) within the targeted brain region, which decreases neuronal activity in that region. High-frequency stimulation (hf-rTMS; 5 Hz or greater) induces long-term potentiation (LTP) and has an excitatory effect (Fitzgerald et al., 2006). The two major forms of synaptic plasticity, LTD and LTP, are NMDA-dependent phenomena that underpin the processes of learning and memory formation (Bear and Malenka, 1994). Hence, rTMS likely promotes brain plasticity, which involves NMDA receptor activation (Huang et al., 2007). Both rTMS modalities produce neurophysiological effects that last longer than the duration of the treatment itself, with the changes induced by excitatory stimulation tending to persist much longer (Janicak et al., 2010). The intensity of the stimulation used during therapy is determined individually for each patient based on the measurement of the motor threshold (MT), which is the minimum strength of the impulse applied to M1 that elicits a muscular response in the fingers (Rothwell et al., 1999). Over the course of many years, the MT was assumed to be a constant value for a given individual. However, recent studies indicate that it fluctuates in a modest, yet significant, range. Cotovio et al. (2021) showed that during antidepressant treatment, MTs varied by up to 5% on average, which should be considered when providing rTMS therapy.

The idea of engaging brain stimulation methods in the treatment of depression stemmed from early neuroimaging studies on brain activity and metabolism, which revealed certain regions of the brain to be particularly involved in the pathogenesis of the disorder. A positron emission tomography (PET) imaging-based study by Baxter et al. (1989) showed significantly decreased cortical activity in the dorsolateral prefrontal cortex (dlPFC) of the left hemisphere in subjects suffering from depression. The dlPFC is strongly connected with the thalamus and basal ganglia (including the caudate nucleus), hippocampus, and primary and secondary association areas (temporoparietal, parietal, and occipital areas). The dlPFC is responsible for higher cognitive functions such as working memory, planning, cognitive control, abstract reasoning, and motor control. Shortly thereafter, another study showed a decreased cerebral blood flow (CBF) within the same area (Bench et al., 1992). Based on those studies, as well as post-stroke reports, Drevets (2000) proposed a theory that hypofrontality is involved in the pathophysiology of depression, and the dlPFC became the main natural target for excitability-enhancing rTMS.

In George et al. (1995) showed significant mood improvement in patients suffering from depression which underwent daily repetitive 20 Hz stimulation administered in 5 sessions for 1 week (*n* = 6). This was the very first study demonstrating rTMS effects on depression symptoms in human subjects, the potential efficacy of rTMS having been suggested by earlier studies on animal models of mood disorders (Fleischmann et al., 1995; Zyss et al., 1997). In O’Reardon et al. (2007) demonstrated that treatment duration impacts treatment effectiveness, and recommended a minimum treatment duration of 4 weeks. This recommendation was included in the official FDA-approved protocol for the treatment of depression, which specified left dlPFC stimulation with rTMS (3,000 pulses, at 10 Hz, –for 37.5 min, at 120% of MT) for 4 up to 6 weeks (Horvath et al., 2010). Since then, the protocol has been extensively studied, resulting in dozens of reports. In Carpenter et al. (2012) published the results from a large study involving 307 subjects. In this study 58% of the patients who underwent the FDA-approved rTMS protocol for TRD achieved symptom reduction, and 37% achieved remission. In 2018, the FDA approved another rTMS protocol targeting the left dlPFC but using a higher frequency in a patterned manner (triplets of pulses at 50 Hz), which allowed a significant reduction in the duration of a single treatment session (3 min) without decreasing its effectiveness (Blumberger et al., 2018). In 2022, the FDA approved another protocol, which was based on intermittent theta-burst frequencies (iTBS). The protocol is called Stanford Accelerated Intelligent Neuromodulation Therapy (SAINT) and involves the delivery of 1,800 iTBS pulses at 90% MT, in less than 10 min, every hour, 10 times a day for 5 days, resulting in 50 treatment sessions equivalent to 90,000 pulses in 5 days. The reported success rate (reduction of symptoms) was 90% at the end of treatment and 60% a month later (Cole et al., 2020). The advantage of this novel protocol over accelerated rTMS is the fact that stimulation is performed using neuronavigation based on functional magnetic resonance imaging (fMRI) results for individual subjects, which has previously been shown to improve the efficacy of treatment (Luber et al., 2017).

The longest follow-up, including measurements over 12 months, showed that the effect of the therapy was sustained over time, with the severity of symptoms after one year equal to that measured immediately after the end of treatment (Janicak et al., 2010). The use of rTMS has also been shown to reduce suicidal ideation by 50%, which should be considered especially important, as in many cases, it could save the patient’s life (Croarkin et al., 2016). Additionally, the method is proving effective in the treatment of depression comorbid with borderline personality disorder, which seems to be of clinical significance, since 41–83% of patients suffering from major depressive disorder are also diagnosed with borderline personality disorder (Ward et al., 2021).

### 2.2. Ketamine as a treatment for depressive disorders

First synthesized in 1963, ketamine is a non-competitive NMDA receptor antagonist that has traditionally been used as a dissociative anesthetic. In 2000, intravenous administration of a subanesthetic dose of ketamine was reported to rapidly reduce depressive symptoms, with this effect being perceptible for up to 72 h (Berman et al., 2000). More extensive studies showed an up to 60–70% efficacy of a single ketamine dose in the treatment of depression (Murrough et al., 2013; Coyle and Laws, 2015; Wilkinson et al., 2018). Of clinical interest is the rapidity of action, with the effect observed as early as 2–4 h after infusion; less comforting is the fact that the therapeutic effect fades rather quickly—within a week after a single administration and about 3 weeks after multiple infusions (Aan het Rot et al., 2010). Actual clinical data analyzed retrospectively, showed a positive response in 44% of patients after six infusions of ketamine (Thomas et al., 2017, 2018). Additionally, ketamine has also been shown to be effective in reducing suicidal ideation and have anti-anhedonic activity (Grunebaum et al., 2018; Wilkinson et al., 2018; Zanos and Gould, 2018). Therefore, not only does ketamine produce rapid and significant symptom relief in patients unresponsive to other antidepressants, but it also appears to have a novel mechanism of action that differs from that of conventional drugs (Smith-Apeldoorn et al., 2022).

## 3. Neuroplasticity—the origin of the healing process

Nearly 80 years ago, Konorski (1948) introduced the world to the concept of brain plasticity, which is the ability of the brain to undergo structural and functional changes in response to internal or external stimuli. Konorski’s pioneering work changed the perception of the nervous system as something immutable, with hundreds of later studies exploring this phenomenon (Konorski, 1948; Merzenich and Sameshima, 1993) Currently, we also have considerable knowledge regarding maladaptive plasticity that occurs in the pathogenesis of mental disorders (Pittenger, 2013; Merzenich et al., 2014). Both rTMS and ketamine have been shown numerous times to promote neuroplasticity, which is likely responsible for their therapeutic effects. Plasticity-related changes are noted at different functional levels of the central nervous system, from changes in neurotransmission and neurotrophic factor levels, through genetic modifications, epigenetic changes, to alterations in brain structure and patterns of brain activity (for review see: Chervyakov et al., 2015; de Vos et al., 2021).

A key role in neuroplastic processes is played by NMDA receptors, which recognizes and binds glutamate, the main excitatory neurotransmitter in the brain. Structurally, NMDA receptors contain a cation channel, which is blocked by a magnesium ion in the resting state. However, cell membrane depolarization tends to displace this magnesium ion, allowing calcium ions to enter the postsynaptic neuron through the, now open, channel. Importantly, both glutamate binding and membrane depolarization are required for NMDA receptors to open, which makes NMDA receptors a coincidence detector. Rapid depolarization eventually leads to LTP induction (Cooke and Bliss, 2006). There are two types of LTP: early and late. Early LTP persists for 30–60 min and is associated with ionic activity and mediator redistribution. Conversely, late LTP can last from a few days up to a few weeks and is associated with gene expression and protein synthesis (Pfeiffer and Huber, 2006; Sutton and Schuman, 2006). NMDA receptors are often located in the same synapse with α-amino-3-hydroxy-5-methyl-4-isoxazolepropionic acid (AMPA) receptors, which also recognize glutamate. The release of glutamate into the synaptic cleft first activates AMPA receptors, resulting in an influx of sodium ions into the cell. The complementary action of NMDA and AMPA receptors is crucial for short-term synaptic plasticity (Hunt and Castillo, 2012; Matveychuk et al., 2020). The function of these receptors undoubtedly plays a role in the therapeutic efficacy of both rTMS and ketamine.

A crucial factor regulating synaptic plasticity is the brain-derived neurotrophic factor (BDNF), which is highly prevalent in the central nervous system, mainly in the hippocampus (Leal et al., 2015). The BDNF supports the existence of nerve cells, promotes the growth and differentiation of new cells and synapses. Its supply is usually reduced in depression (Dwivedi, 2009).

### 3.1. rTMS-induced neuroplasticity

There are three main categories of rTMS-induced changes in neuroplasticity. They involve: brain function and structure, neurotransmission, and neurotrophic factor levels. In addition to exclusive neuroplastic modifications, rTMS effects on anti-inflammatory actions have been also observed. Certain effects have been documented in humans, while others have been demonstrated primarily in animal models (Chervyakov et al., 2015).

Several indicators of rTMS-induced neuroplastic changes in humans have been identified so far. The results described below relate to the treatment of depression only. For instance, Kito et al. (2012) demonstrated that 2 weeks of 10 Hz rTMS over the left dlPFC significantly increased CBF within the stimulated area. This increase represents a reversal of the previously identified decrease in CBF associated with depressive disorder (Bench et al., 1992). More evidence for functional plasticity induced by rTMS comes from a recent paper on fMRI published by Eshel et al. (2020). Those authors observed an increase in overall global connectivity of the dlPFC to other brain structures, and a significant decrease in the strength of its connectivity with the amygdalae after 4 weeks of daily 10 Hz rTMS treatments. The rTMS-induced increase in prefrontal cortex activity and suppression of excessive limbic cortex activity correlates with the effects observed on the mental level, i.e., improved mood, reduced anxiety, and better cognitive functioning. Seewoo et al. (2022) demonstrated that 6 weeks of 10 Hz rTMS also increased cortical volume within the dlPFC, as well as a few other frontal areas, further proving that rTMS induces structural plasticity. When considering neurotransmission, Pogarell et al. (2007) demonstrated that 10 Hz rTMS applied to the left dlPFC increases dopamine levels in the striatum. Additionally, Luborzewski et al. (2007) noted an elevation in the level of glutamate within the targeted area. Moreover, a recent study by Spurny-Dworak et al. (2022) showcased an increase in glutamate transmission also induced by facilitatory TMS (iTBS, intermittent theta-burst stimulation). Importantly, both neurotransmitters are deficient in depression. Regarding neurotrophic factors, Yukimasa et al. (2006) showed that 10 sessions of 20 Hz rTMS raise peripheral blood levels of BDNF.

In animal models, rTMS has shown promising results in reversing depressive-like behaviors and modulating key neurobiological mechanisms. For example, in rodents, rTMS was found to increase neurogenesis and synaptogenesis in the hippocampus and normalize synaptic plasticity in the prefrontal cortex, as well as enhance glutamate release and signaling within both areas (Ramírez-Rodríguez et al., 2022; Qian et al., 2023). It was also demonstrated that hf-rTMS can upregulate BDNF expression in the hippocampus and prefrontal cortex (Gersner et al., 2011). Additionally, studies in primates showed that rTMS treatment can promote the proliferation and survival of neural stem cells in the hippocampus, leading to an increase in the number of newly formed neurons (Perera et al., 2007). Regarding rTMS influence on inflammatory factors, Zuo et al. (2022) demonstrated in mice subjected to the chronic unpredictable stress procedure that hf-rTMS not only significantly alleviated the activation of microglia but also induced a shift in microglial polarization from the pro-inflammatory to the anti-inflammatory phenotype in both the hippocampus and prefrontal cortex. Simultaneously, rTMS reversed the stress-induced decrease in astrocyte numbers and suppressed the elevated concentrations of interleukin (IL)-6, IL-1β, and tumor necrosis factor-alpha (factors extensively studied in relation to the pathogenesis of depressive disorders) in the aforementioned brain regions.

Data from animal models allow us to explore the issue far more deeply than data from human subjects; however, extrapolating the results of cellular and molecular approaches as though they also applied to humans should be done with caution. The feasibility of developing animal models of mental disorders is a matter of debate (Belzung and Lemoine, 2011; Baker et al., 2020). Furthermore, the electromagnetic coil used to generate an impulse of sufficient strength is often much larger than the head of a rodent (the most frequent experimental subject). Therefore, when studying rodents, it is difficult to postulate that the rTMS impulse selectively stimulates some specific area of the brain, and we must assume that the whole organ is stimulated (Beom et al., 2016; Tang et al., 2017).

### 3.2. Ketamine-induced neuroplasticity

The mechanisms of action of ketamine, like the effects of rTMS, can be described on several levels, which—apart from structural or functional plasticity on the network level—encompass the molecular and cellular alterations associated with synaptic plasticity and shifts in neurotransmission.

The few neuroimaging studies on the response of a human brain to ketamine treatment have revealed several noteworthy changes. One study by Lehmann et al. (2016) demonstrated, based on fMRI data, an increased activity in the anterior cingulate cortex (ACC) in healthy subjects following a single ketamine infusion. Another study showed an increased global functional connectivity in prefrontal cortices in depressed subjects after a single dosage of ketamine, which suggests the involvement of the glutamatergic system (Abdallah et al., 2018). Moreover, structural MRI data showed increased hippocampal and decreased nucleus accumbens (NAc) volumes. The changes correlated with symptomatic improvement 1 day after a single infusion (Abdallah et al., 2017). Furthermore, Evans et al. (2018) demonstrated normalization of the functional connectivity between the insular cortex [part of the salience network (SN)], and the default mode network (DMN) 2 days following a single ketamine infusion; however, this effect was no longer present in a subsequent measurement 8 days later. Kotoula et al. (2023) presented a very detailed and insightful review on the brain changes accompanying TRD and on TRD treatment with rTMS, ketamine, and other methods.

A meta-analysis of studies on various NMDA antagonists, including traxoprodil, lanicemine, and rapastinel, suggests that their potency in reducing depressive symptoms is weaker than that of ketamine, and their effects are insufficient to achieve remission (Kishimoto et al., 2016). Those findings clearly indicated that ketamine’s antidepressant mechanisms of action extend beyond merely blocking NMDA receptors.

The bulk of the research on the molecular and cellular mechanisms of ketamine’s action comes from animal models, which provide valuable insights into the therapeutic efficacy of this NMDA receptor antagonist. The antidepressant effect of ketamine likely involves a cascade of sequential events (Aleksandrova et al., 2017; Yang et al., 2019). Ketamine has a higher affinity for NMDA receptors located on inhibitory γ-aminobutyric acid (GABA) interneurons, which normally suppress glutamatergic excitation. Thus, ketamine prevents the activation of GABA interneurons, leading to increased glutamate levels. Consequently, higher glutamate levels initiate the activation of postsynaptic AMPA receptors, resulting in the enhancement of mammalian target of rapamycin (mTOR) and BDNF signaling pathways. This process ultimately augments synaptic connection strength and synaptic plasticity (Zhang et al., 2021; Xu et al., 2022). Furthermore, studies have shown that ketamine administration increases electrophysiological signals associated with AMPA receptor transmission in the hippocampus and prefrontal cortex region (Björkholm et al., 2015; El Iskandrani et al., 2015). Ketamine was also found to increase BDNF and mTOR expression in the hippocampus (Yang et al., 2013). Conversely, administration of an AMPA receptor antagonist suppresses both BDNF and mTOR signaling pathways and nullifies the behavioral effects of ketamine therapy (Zhou et al., 2014).

Beyond the molecular and cellular level, ketamine also affects monoaminergic neurotransmission. Firstly, it increases glutamatergic transmission to midbrain neurons by disinhibiting the prefrontal cortex. Secondly, it enhances serotonergic transmission via the projection of pyramidal neurons from the prefrontal cortex to the sutural nucleus. Finally, it stimulates glutamate release, thereby enhancing neurotransmission in the prefrontal cortex of the rat brain (Moghaddam et al., 1997; Zanos et al., 2018). Furthermore, ketamine induces 5HT1b receptor upregulation in the NAc, ventral globus pallidus, and nucleus reuniens in the thalamus. It also inhibits serotonin transporter activity in both the prefrontal cortex and the midbrain (McIntyre et al., 2021). The effects of ketamine extend to dopaminergic and noradrenergic transmission, with an increased dopaminergic and noradrenergic activity observed in regions including the prefrontal cortex, striatum, NAc, ventral tegmental area, and locus coeruleus. Ketamine additionally downregulates metabotropic glutamatergic receptor 5 (mGluR5), and it is high mGluR5 levels that are associated with antidepressant effects. Another noteworthy effect of ketamine involves the elevation of vascular endothelial growth factor, which is crucial for the neurotrophic effects of BDNF (Tian et al., 2022). Intriguingly, ketamine has been stipulated to reverse the hypoconnectivity in the prefrontal cortex and hippocampus often observed in depressive disorders. Moreover, ketamine could mitigate the hyperconnectivity observed in the NAc as well. This effect might shift the balance of neural activity from limbic and subcortical structures toward the cortex, notably the prefrontal cortex. This transition potentially results in the attenuation of limbic responses to negative emotional stimuli (Mihaljević et al., 2020).

## 4. Combined rTMS and ketamine treatment

**FIGURE 1:**
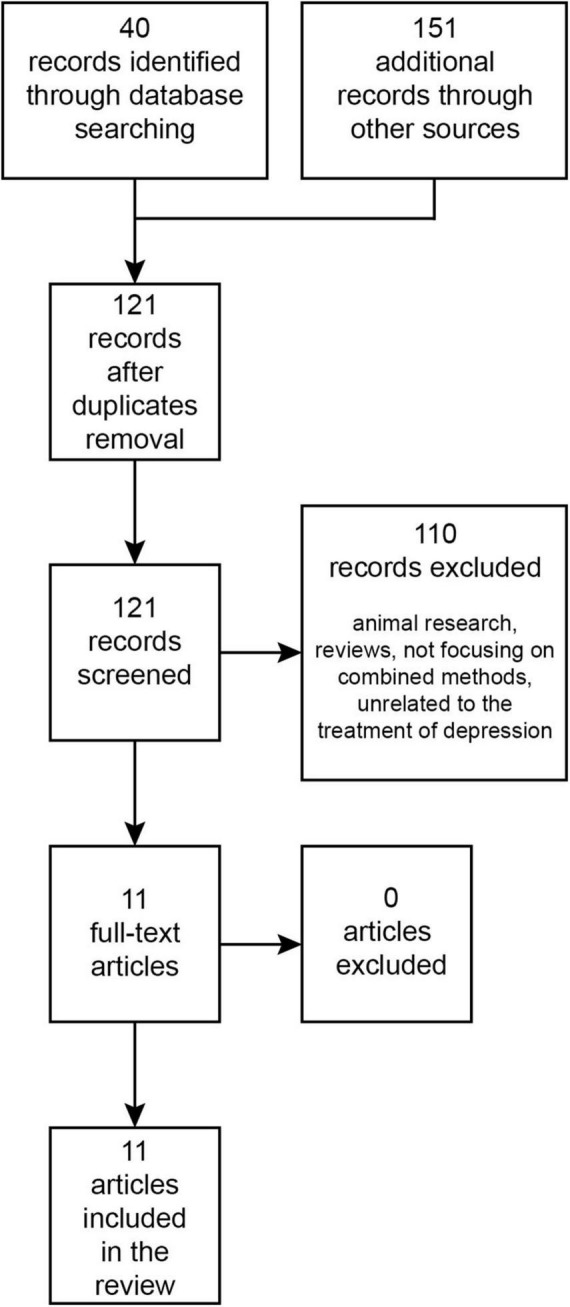
Diagram illustrating the structured approach to literature research.

The PubMed/MEDLINE and Web of Science databases were searched from their inception until September 2023 for research papers written in English using the terms: “transcranial magnetic stimulation,” “ketamine,” “esketamine,” “combined/combining,” and “depression.” The Boolean search query used was: [(combin* “Transcranial Magnetic Stimulation” AND ketamine) OR (combin* “Transcranial Magnetic Stimulation” AND esketamine) OR (combin* rTMS AND ketamine) OR (combin* rTMS AND esketamine)] AND depress*. This initial search in databases returned 40 records. Additionally, an auxiliary search was performed on Google Scholar, which produced 151 records. Once duplicates were eliminated, 121 records were retained. After reviewing titles and abstracts, 110 papers were set aside, leaving 11 papers for full-text examination. All of these were incorporated into the review (Figure 1). To our best understanding, these cover all existing publications on this topic.

The concept of combining rTMS and ketamine treatments is not novel, yet still very few studies exist describing such combined treatments. Furthermore, the few publications that address the combined treatment vary significantly in terms of treatment protocols and methodology. Since they are pioneering works, they will be briefly characterized in chronological order (for a summary of the key attributes from the referenced studies see Table 1).

**TABLE 1:** Summary of key features and findings of the reports describing the combination of rTMS and ketamine in the treatment of depression.

References	Type of study	Diagnosis	Number of subjects	Intervention (combination of TMS + ketamine)	rTMS protocol: frequency/duration of single session/stimulated area	Ketamine dosage/infusion duration	Effect of treatment
Best, 2014a	Case-study	Bipolar disorder	*N* = 1	Concurrent; once a week for 3 years	1 Hz, 115% MT, 40 min, ACC	40–80 mg IV; 30 min	Significant reduction in symptoms with functional improvement
Best, 2014b	Retrospective study	Unipolar/bipolar disorder	*N* = 28	Concurrent; from 6 to 55 treatments, frequency of treatments was not given	1 Hz, 115% MT, approximately 60 min, ACC	Increasing from 20 to 300 mg IV, 20–120 min	Reduction in symptoms with substantial functional improvement
Best and Griffin, 2015	Case-study	Unipolar disorder	*N* = 1	Concurrent; once a week for 13 weeks	1 Hz, 115% MT, 40 min, ACC	Increasing from 30 to 100 mg IV; 30 min	Substantial decreases in depression, suicidal ideation, alcohol use, and concentration difficulties along with increased sense of self-purpose
Best et al., 2015	Case-study	Bipolar disorder	*N* = 1	Concurrent; approximately once a week for 5 months (a total of 24 treatments)	1 Hz, 115% MT, 40 min, ACC	Increasing from 50 to 600 mg IV, 30 min	Substantial decreases in both depression and mania symptoms
Best, 2015	Case-study	Unipolar disorder comorbid with OCD	*N* = 1	Concurrent; twice a week for 10 weeks	1 Hz, 115% MT, 50 min, ACC	Increasing from 40 to 425 mg, 40 min	Significant improvement in depression symptoms and reduced anxiety; lack of effectiveness for OCD
Best and Pavel, 2017a	Case-study	Unipolar disorder	*N* = 1	Concurrent; once a week for 5 months	1 Hz, 115% MT, 40 min, ACC	Increasing from 50 to 250 mg IV, 30 min	Significant clinical improvement in depressive symptoms, anxiety and suicidality
Best and Pavel, 2017b	Case-study	Unipolar disorder	*N* = 1	Concurrent; once a week for 4 months	1 Hz, 115% MT, 40 min, ACC	Increasing from 50 to 250 mg IV, 30 min	Long-term significant reduction in symptoms of depression and anxiety
Best et al., 2019 *	Retrospective study	Unipolar/bipolar disorder	*N* = 28	Concurrent; three times a week (a total of 10–30 treatments)	1 Hz, 130% MT, 30 min, ACC	Starting from 20 mg with an average dosage range of 0.4–2.3 mg/kg IV, 20 min	Long-term significant reduction in depressive symptoms
Elkrief et al., 2022	Case-study	Bipolar disorder	*N* = 1	Non-concurrent; initial phase (2 weeks): 3 iTBS sessions per day; twice a week. consolidation phase (10 weeks): 48x cTBS, 48x, iTBS (3 daily session twice a week for 6 weeks, then once a week); 8 intramuscular ketamine injections (once a week for 6 weeks, then every 2 weeks)	Initial phase: iTBS (600 pulses per session), 120% MT, left dlPFC Consolidation phase: cTBS (600 pulses per session), 120% MT, right dlPFC; iTBS (600 pulses per session), 120% MT, left dlPFC	Initial phase: 0.5 mg/kg IV; consolidation phase: 0.5 mg/kg intramuscular, split into two dosages given 30 min apart	Complete and sustained remission
Best et al., 2022	Retrospective case-series study	Unipolar/bipolar disorder comorbid with other psychiatric conditions	*N* = 4 (treated with rTMS + ketamine out of the total *N* = 6)	Concurrent; from 10 to 30 treatments; frequency of treatments was not given	1 Hz, 130% MT, 30 min, ACC	Starting from 20 mg with an average dosage range of 0.4–2.3 mg/kg IV, 20 min	Significant clinical improvements in symptoms
Payette et al., 2023	Retrospective study	Unipolar disorder	*N* = 21	Non-concurrent; 6 ketamine injections over 2 weeks preceded with rTMS	10 Hz, 120%, left dlPFC	0.5 mg/kg IV over 60 min	19% responded, 9.5% achieved complete remission

ACC, anterior cingulate cortex; cTBS, continuous theta-burst stimulation; dlPFC, dorsolateral prefrontal cortex; Hz, hertz; min, minutes; iTBS, intermittent theta-burst stimulation; IV, intravenous; OCD, obsessive-compulsive disorder; rTMS, repetitive transcranial magnetic stimulation; MT, motor threshold; *, indicates a follow-up study to Best (2014b).

The very first report on the combined use of rTMS and ketamine (Best, 2014a) is a case-study of a severely depressed 31-year-old male diagnosed with bipolar disorder who did not achieve significant improvement with prior ECT, rTMS, vagal nerve stimulation, or pharmacotherapy. The combined treatment was conducted once a week for 3 years. A 30-min intravenous infusion of ketamine was administered during each rTMS treatment (1 Hz at 115% MT for 40 min), with 5 min of sole stimulation before and after the superimposed ketamine infusion. rTMS was applied to the medial prefrontal cortex, which corresponds to the ACC. A decreased activation of this region is associated with depressive symptoms. Reportedly, the patient achieved a partial but significant symptom reduction and functional improvement.

A further paper by Best (2014b) is a retrospective study involving 28 cases of TRD in patients with either unipolar or bipolar affective disorders. Twenty of those patients underwent a pretreatment phase lasting anywhere from 3 days to 2 weeks. This involved intensive rTMS treatment, administered three times a day for 6 or 7 days per week, or a priming TMS session immediately before the combined TMS-ketamine infusion therapy. In contrast, eight patients did not receive any form of pretreatment or priming. The number of rTMS treatments with simultaneous ketamine infusion varied among patients. In all patients rTMS at 1 Hz was applied to the ACC, and the ketamine dose was increased in subsequent treatments. Notably, all 28 patients who completed the initial month of treatment not only experienced alleviation of their psychiatric symptoms but also demonstrated marked psychosocial improvement.

Another case-study by Best and Griffin (2015) showed a significant decrease in depression symptoms in a 23-year-old woman who had failed to achieve improvement with standard pharmacotherapy received over the previous 9 years. As part of the study the woman received a combined rTMS and ketamine therapy (lf-rTMS applied to the ACC). Thirty-minute stimulation sessions were conducted during an infusion of ketamine once a week for 13 weeks.

Subsequently, a case-study by Best et al. (2015) described the case of a man suffering from drug-resistant bipolar affective disorder. Initial evaluations included a psychometric assessment and a single-photon emission computed tomography (SPECT) scan of the brain. The psychometric results indicated pronounced depressive and manic symptoms, consistent with a diagnosis of bipolar disorder. The SPECT findings mirrored the psychometric data. Over the span of five months, the patient underwent combined rTMS and ketamine treatment 24 times. The initial ketamine dose of 50 mg was gradually increased to 600 mg by the final session; lf-rTMS was applied to the ACC. Notably, the patient began reporting marked symptom relief after the second session. Five months post-treatment, a follow-up psychometric evaluation and SPECT scans showed notable increases in blood flow in areas previously identified as deficient.

Another case-study by Best (2015) documented the efficacy of combining rTMS and ketamine in treating TRD comorbid with obsessive-compulsive disorder (OCD). Consistent with other studies, lf-rTMS was applied to the ACC alongside concurrent ketamine infusions, administered twice weekly for 10 weeks. While the treatment led to a significant reduction in depressive symptoms, it did not notably impact OCD symptoms.

Two additional case-studies by Best and Pavel (2017a,b) involved two female patients with drug-resistant unipolar or bipolar depression. These studies employed the same procedure of simultaneous lf-rTMS stimulation targeted at the ACC and infusions of ketamine at escalating doses. The outcomes were consistent for both patients, with each achieving not only a significant long-term reduction in depression and anxiety symptoms, but also marked functional improvement and elevated quality of life. These clinical improvements were further supported by notable increases in cortical and subcortical blood flow, as revealed by brain SPECT imaging.

In another study by Best et al. (2019), a follow-up of Best (2014a) was presented. The authors propose that the synergistic effect of ketamine with rTMS stems from the fact that, as an analgesic, ketamine permits the use of electromagnetic pulses of greater strength, thus improving the therapeutic effect. Unfortunately, the study was a retrospective analysis which, along with the lack of a control group, makes it not very reliable and thus not sufficient to make a clear statement on the matter.

Elkrief et al. (2022) reported a case-study of a 43-year-old male diagnosed with bipolar disorder and suffering from severe TRD. The initial phase lasting 2 weeks involved iTBS (600 pulses per session/3 sessions per day) applied to the left dlPFC and intravenous ketamine (0.5 mg/kg) infusions two times a week. The consolidation phase, which lasted 10 weeks, involved iTBS to the left dlPFC and continuous TBS to the right dlPFC (600 pulses per session in both cases, 48 sessions in total) and 8 doses of ketamine administered intramuscularly (once per week for the first 6 weeks, then once every 2 weeks). The patient achieved full and sustained remission; however, it is worth noting that the iTBS protocols used in this study diverge from those used in the other analyzed studies, where lf-rTMS was the primary method of stimulation.

A recent study by Best et al. (2022) verified the utility of SPECT imaging in evaluating the efficacy of therapeutic modalities. This includes the procedure of simultaneous rTMS inhibitory stimulation in combination with ketamine infusions, a method that these authors have repeatedly investigated. Symptom reduction was observed in all patients. The study concluded that the described imaging modality serves as a useful complementary diagnostic tool.

The last paper on the combined use of rTMS and ketamine explores a less direct interaction between the two methods. In the study by Payette et al. (2023), ketamine therapy was administered to patients who did not experience positive outcomes from rTMS therapy. Twenty-one patients with TRD received six ketamine infusions immediately following an unsuccessful four-week rTMS protocol (10 Hz rTMS targeting the left dlPFC). Such a treatment led to the reduction of symptoms in 33% of the patients. It is tempting to speculate that the reduction of symptoms could have been partially linked to the previous rTMS treatment, as even if it did not result in a quantifiable reduction in the severity of symptoms, it does not mean that it left the central nervous system without any changes of a neuroplastic nature. However, it’s crucial to recognize that this study was a retrospective analysis and not a comprehensive research project, which challenges the drawing of definitive conclusions and the complete trust in the validity of the results.

To sum up, as of yet, there have been only a few studies investigating the effect of a combined rTMS and ketamine protocol on depressive symptoms. Most of the studies included neither a control group nor a sufficient number of subjects (mostly case reports) and were primarily retrospective (Table 1). Moreover, aside from Elkrief et al. (2022), the studies lack sufficient justification for the specified treatment parameters i.e., the stimulation site for rTMS, stimulation frequency, and the duration and number of sessions. Given the fact that 15 years have elapsed since the official approval of rTMS and 4 years since the first endorsement of (S)-ketamine use, it is surprising that a comprehensive analysis of their potential synergistic effects has not been conducted. Further experimental work is needed to address this knowledge gap, particularly as many clinicians around the world are already offering combined rTMS and ketamine therapy to their patients.

## 5. Summary

All available studies on the use of combined rTMS and ketamine for treating depressive symptoms have been considered in this review. Regrettably, the number of these studies is limited, and there are no randomized controlled trials (RCTs) among them. The individual studies also present significant methodological discrepancies, which precludes drawing any consistent conclusions regarding the precise use of combined rTMS and ketamine. Despite these limitations, the analyzed studies provide persuasive evidence that supports further exploration of these two treatment modalities combined, at the same time highlighting an imperative for more rigorous research. A noteworthy gap exists in the current literature, as no experimental work has delved into the neurobiological underpinnings of the combined rTMS and ketamine treatment. Exploring this is not only academically intriguing but also crucial for refining clinical applications.

### 5.1. Combined treatment of rTMS and ketamine: future perspectives

Both rTMS and ketamine stand as evidence-based methodologies, substantiated through rigorous scientific scrutiny, showcasing efficacy in the management of TRD. In a direct comparison, both treatment modalities demonstrate similar effectiveness, with high remission and response rates (Mikellides et al., 2022).

**FIGURE 2:**
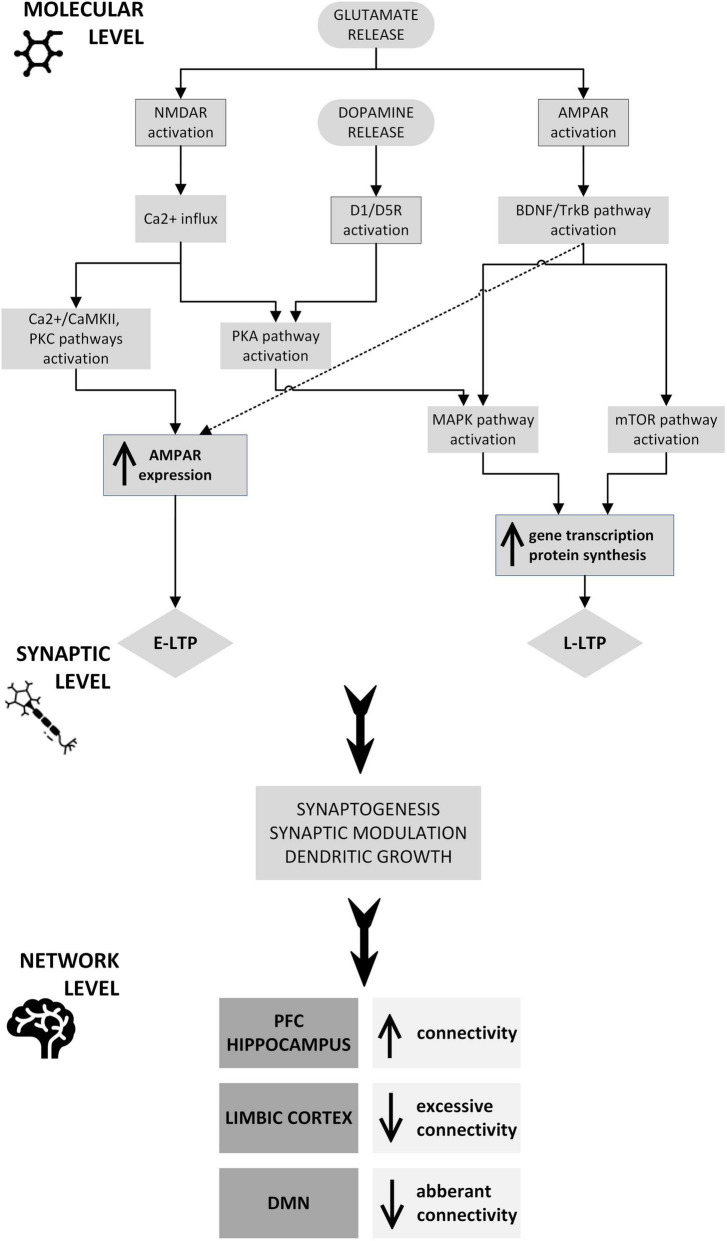
Potential neuroplastic mechanisms underlying the therapeutic and synergistic effects of combined rTMS and ketamine treatment: rTMS and ketamine induce glutamate release, which at a molecular level causes NMDAR and AMPAR activation through signaling pathways, including CaMKII, PKC, PKA, BDNF/TrkB, MAPK, and mTOR. The CaMKII and PKC pathways, as well as the TrkB pathway, stimulate AMPAR trafficking into the postsynaptic neuron membrane, which is one of the mechanisms of E-LTP. PKA, BDNF/TrkB, MAPK, and mTOR pathway activation induces modulation of plasticity-related gene transcription and protein synthesis. These processes underlie L-LTP development. The expression of genes that are essential for synaptic plasticity may also be stimulated by dopamine activation of D1R, D3R, or both, through PKA and MAPK pathways. This intracellular process results in synaptogenesis, synaptic modulation, and dendritic growth, leading to connectivity changes in the PFC, hippocampus, limbic cortex, and DMN. AMPAR, α-amino-3-hydroxy-5-methyl-4-isoxazolepropionic acid receptor; BDNF, brain-derived neurotrophic factor; CaMKII, Ca2+/calmodulin-dependent protein kinase; D1/D5R, dopamine receptor 1/5; DMN, default mode network; E-LTP, early long-term potentiation; L-LTP, late long-term potentiation; MAPK, mitogen-activated protein kinase; mTOR, mammalian target of rapamycin; NMDAR, N-methyl-D-aspartate receptor; PFC, prefrontal cortex; PKA, protein kinase A; PKC, protein kinase C; TrkB, tropomyosin receptor kinase B.

Repetitive transcranial magnetic stimulation (rTMS) uses an electromagnetic field to modulate the activity of various structures and circuits in the brain by stimulating a specific area, while ketamine is an NMDA receptor antagonist whose effects include changes in the glutamatergic system. These two treatments have different modes of action and target brain pathways differently, acting in a complementary manner. Both rTMS and ketamine are known to promote neuroplasticity, the brain’s ability to reorganize and form new connections, which undoubtedly plays a role in recovery from mental health disorders. Each method individually affects synaptic activity, induces changes in neurotransmission, and influences various neuronal circuits to produce an effect at the network level. The use of combined rTMS and ketamine in treatment may potentiate the effect of either method alone through mutual reinforcement, leading to a more significant and lasting therapeutic effect and giving stronger and faster improvement than the methods used separately. Firstly, the increased effectiveness of combined methods is likely based on increased glutaminergic and dopaminergic transmission. Secondly, BDNF expression is elevated peripherally and in specific cortical and subcortical areas. Finally, increased activity in certain brain structures, including the dlPFC, coupled with decreased activity in the limbic regions, particularly the amygdala, may normalize functional connections and restore the balance disrupted by the prolonged presence of depressive disorders. Thus, it seems reasonable to conclude that the mechanism of action/potentiation is probably centered around neuroplasticity (Figure 2).

Numerous studies have identified various factors that may be involved in the onset and development of depressive disorders (for a review see: Blackburn, 2019). The first instance of these is the biochemical disruption of monoamines and their receptors (Coppen, 1967). It is assumed to be a naturally occurring and transient phenomenon in mood disorders. Nonetheless, relatively recently the hypothesis of a neuroprogressive nature of depression has emerged (Ruiz et al., 2018). The hypothesis acknowledges a broader context of the etiology of the disorder and defines it as a dangerous condition resulting from or involving a significant impairment of brain function, and more specifically, neuronal deterioration involving increased apoptosis, impaired neurogenesis, synaptogenesis, and even loss of synapses and dendritic arborization. This results in decreased neuroplasticity and an increased immune response. Neuronal atrophy in the prefrontal and limbic regions was shown to be is associated with low levels of BDNF and other neurotrophic factors (Price and Duman, 2020). Considering that depression is a result of disruption of the brain’s neuroplasticity processes, the use of treatment involving a combination of two methods that intensify or even restore these processes seems noteworthy. Hence, in light of the described mechanisms of a likely mutual reinforcement of effects, combined rTMS and ketamine may become a future treatment for TRD and perhaps other mental disorders as well. This hypothesis has been already proposed by Kuo et al. (2023).

### 5.2. Safety of a combined rTMS and ketamine treatment

There are no reports of harmful effects of using a combination of the two methods. It was shown that both racemic and S-ketamine enhance motor cortical excitability, which must raise caution in the context of combining excitatory rTMS stimulation with simultaneous ketamine treatment (Di Lazzaro et al., 2003; Höffken et al., 2013). However, as revealed by the study by Ramakrishnan et al. (2019), such an effect was not observed in the prefrontal cortex. Four hours after 0.5 mg/kg ketamine infusion, the excitability of the dlPFC was lower than before, and after 24 h, it returned to normal but remained lower than baseline. The authors emphasize that this was a preliminary study, and the results need to be confirmed in a larger sample. Nevertheless, if it is assumed that ketamine does not increase excitability within the prefrontal cortex, then applying excitatory rTMS to the dlPFC could be done safely without the risk of triggering a rare yet most serious side effect, which is a seizure. Moreover, although care and caution should be exercised, asynchronous application of treatments may be assumed to remain safe. However, to enhance treatment safety and efficacy, it is necessary to optimize the intensity of treatment, particularly in terms of its duration and the number of rTMS treatments and ketamine doses needed, based on clinical evaluation.

The potential synergy of rTMS and ketamine is a promising sign for a combined treatment approach. Drawing from numerous studies on the application of rTMS and ketamine for TRD treatment (e.g., Lefaucheur et al., 2020; Alnefeesi et al., 2022), as well as previous findings on the combined use of these methods, there is a likely benefit in implementing full protocols of both methods in a parallel, yet non-simultaneous, manner. The robustness of this hypothesis necessitates further rigorous investigation in the future.

### 5.3. Considerations and precautions of combining rTMS and ketamine in depression treatment

In the evolving landscape of treating depressive disorders, the combination of rTMS and ketamine represents a novel pathway that leverages the unique benefits of both treatment modalities. While both treatments individually have shown promise, their combined use is in the nascent stages of research and application. This necessitates a meticulous approach toward understanding the precise considerations and precautions necessary to optimize patient outcomes while safeguarding their wellbeing. Here, we outline the essential guidelines that practitioners should keep in mind when navigating the path of combined treatment.

Harnessing the synergistic effects of rTMS and ketamine potentially elevates the effectiveness of these therapeutic strategies, fostering improved patient outcomes. Central to this approach is the formulation of personalized treatment strategies, meticulously crafted based on a deep understanding of individual patient variables, including medical history and the present severity of depression—a tactic designed to ensure the most suitable and responsive treatment pathway. This bespoke approach should be complemented by a regime of continuous monitoring, enabling healthcare providers to keenly observe patient responses and make necessary adjustments in real time, thereby fostering optimal treatment outcomes through proactive management. This strategy advocates for a patient-centered approach leveraging the potential enhanced benefits from the combined use of rTMS and ketamine in depression management.

Given the increased scrutiny required when navigating the combined use of rTMS and ketamine, ensuring patient safety through stringent precautions is non-negotiable. A primary concern is managing the augmented side effects, necessitating a comprehensive strategy for their monitoring and adept handling. A detailed psychiatric evaluation, which meticulously examines any history of psychosis, mania, or substance abuse, is essential in avoiding potential complications. In-depth analysis of the given patient’s medical history, especially focusing on their cardiovascular and neurological health, is pivotal in identifying contraindications. Although, both rTMS and ketamine have their own contraindications (Rossi et al., 2009, 2021; Molero et al., 2018; Smith-Apeldoorn et al., 2022), there is a considerable overlap, namely: (a) history of seizures or epilepsy: both rTMS and ketamine should be used with caution or avoided in individuals with a history of seizures or epilepsy (it is advisable to conduct an electroencephalographic examination prior to treatment), (b) medical conditions that significantly affect brain structure or function, such as brain tumors, significant cerebrovascular disease, or metal/electronic implants in the head (with the last one being especially contraindicated for rTMS), (c) current substance abuse or addiction: both treatments may not be suitable for individuals with active substance abuse or addiction issues; ketamine has abuse potential, and rTMS efficacy may be compromised in those with ongoing substance use, (d) certain medications: some medications may interact with either rTMS or ketamine, or influence their effectiveness, (e) mental instability: rTMS and ketamine are typically not recommended for individuals with severe mental instability (active psychosis or a high risk of self-harm), (f) uncontrolled hypertension: high blood pressure that is not well-controlled may pose a risk as both methods can affect blood pressure, (g) pregnancy: both treatments are typically avoided during pregnancy unless the potential benefits surpass the risks.

Similarly, both rTMS and ketamine have the potential to cause side effects (Molero et al., 2018; Rossi et al., 2021; Smith-Apeldoorn et al., 2022); those that have been reported for either method alone include: (a) lightheadedness or dizziness during or after the procedure, (b) insomnia or sleep disturbances, (c) mania or hypomania: although rare, both rTMS and ketamine treatments have been associated with inducing manic or hypomanic states, particularly in individuals with bipolar disorder, (d) seizures (extremely rare): there is an extremely low risk of seizures, especially in individuals with a history of epilepsy or seizures.

To maintain patient safety during the treatment phase, adherence to well-defined safety and emergency protocols is essential, with the support of a quick-response team ready for emergencies. Encouraging open communication about the experimental nature of the dual treatment and guiding patients through a detailed consent process is vital. It sets a foundation of transparency, presenting the potential risks and benefits clearly. Furthermore, establishing an emergency preparedness plan ensures a controlled environment during treatment.

To conclude, it is essential to maintain a visionary stance that facilitates regular patient follow-ups while actively engaging with the rapidly progressing research landscape in this sector. This fosters a treatment trajectory that is progressively grounded in empirical insights.

### 5.4. Limitations

The manuscript at hand endeavors to establish a theoretical framework for the combined use of rTMS and ketamine in antidepressant treatments, hypothesizing a superior efficacy of the combined treatment compared with that of each method used alone. This is a particularly compelling area of research, given the rising global burden of depression and the continuous search for more effective treatment modalities.

Given the paucity of research in this specific area and the methodological shortcomings of existing studies, a meta-analysis or a comprehensive critical review remains unfeasible at this juncture. These facts prove this interdisciplinary inquiry is still in its infancy and highlight the need for increased investment in quality research to bridge these gaps.

Nevertheless, we have exerted meticulous efforts to precisely delineate the outcomes derived from an independent use of each method and to explore potential intersections and synergies. This exploration is not only crucial for clinicians who opt for tailored treatment regimens but also offers insights into potential mechanisms at play when both interventions are combined.

Undeniably, there is a pressing need for rigorously designed studies featuring appropriate control groups and well-defined conditions to pave the way for a standardized protocol combining rTMS and ketamine treatments. To truly revolutionize the treatment landscape, it is imperative that we also understand the nuances and intricacies of patient responses. Furthermore, a deep dive into the neurobiological underpinnings governing the mechanisms of action of this combined approach is warranted. Such insights would not only demystify the observed therapeutic effects but also pave the way for refining and optimizing the protocols for maximum efficacy and safety.

## Author contributions

WD: Writing – original draft. MW: Writing – review and editing. MD: Writing – review and editing. ZK: Writing – review and editing. PM: Writing – review and editing. AS: Supervision, Writing – review and editing.
