# Corrigendum: Transcranial magnetic stimulation and ketamine: implications for combined treatment in depression

**DOI:** 10.3389/fnins.2024.1507512

**Published:** 2024-10-23

**Authors:** Weronika Dębowska, Magdalena Więdłocha, Marta Dębowska, Zuzanna Kownacka, Piotr Marcinowicz, Agata Szulc

**Affiliations:** ^1^Department of Psychiatry, Faculty of Health Sciences, Medical University of Warsaw, Warsaw, Poland; ^2^KeyClinic, Warsaw, Poland; ^3^MindHealth, Warsaw, Poland

**Keywords:** depressive disorders, treatment-resistant depression, transcranial magnetic stimulation, TMS, rTMS, ketamine, combined TMS and ketamine, neuroplasticity

In the published article, there was an error regarding the affiliations for Magdalena Więdłocha, Piotr Marcinowicz, and Agata Szulc. As well as having affiliation 1, Magdalena Więdłocha and Piotr Marcinowicz should also have an additional affiliation 2: KeyClinic, Warsaw, Poland. As well as having affiliation 1, Agata Szulc should also have an additional affiliation 3: MindHealth, Warsaw, Poland.

In the published article, there was an error in the Conflict of interest statement. The original Conflict of interest statement was: “The authors declare that the research was conducted in the absence of any commercial or financial relationships that could be construed as a potential conflict of interest.” It did not include all of the authors' affiliations and potential financial conflicts of interest.

Two co-authors of the article (MW and PM) were employed by KeyClinic—a commercial mental health center which provides ketamine and TMS treatment, as one of many services. The center offers multiple psychiatric and psychotherapeutic services and does not focus on ketamine/TMS treatment or market it in any way. Moreover, one of the authors (AS) was employed by Mind Health (a commercial medical center), was a member of Janssen Cilag Advisory Board and gave lectures for Janssen Cilag.

The correct Conflict of interest statement appears below.

